# Evodiamine Boosts AR Expression to Trigger Senescence and Halt Proliferation in OSCC Cells

**DOI:** 10.3390/cimb47070558

**Published:** 2025-07-17

**Authors:** Gang Chen, Hong-Liang Du, Jia-Nan Liu, Jie Cheng, Jing Chen, Xiao-Yang Yin, Hu-Lai Wei, Jing Wang

**Affiliations:** 1 School of Basic Medical Sciences, Lanzhou University, Lanzhou 730000, China; cheng19@lzu.edu.cn (G.C.);; 2 The First Hospital of Lanzhou University, Lanzhou 730000, China; duhongliang2022@sina.com; 3 General Practitioner Primary Training Base of the First Hospital of Lanzhou University, Lanzhou 730000, China; 4 Hospital of Stomatology, Lanzhou University, Lanzhou 730000, China

**Keywords:** evodiamine, androgen receptor, oral squamous cell carcinoma, senescence

## Abstract

Oral squamous cell carcinoma (OSCC), an aggressive and poorly prognosed subtype of head and neck squamous cell carcinoma (HNSCC), has prompted urgent calls for innovative therapeutic approaches. Evodiamine (EVO), a natural alkaloid extracted from the Chinese herb *Evodia rutaecarpa*, has demonstrated significant potential in curbing tumor cell proliferation and slowing tumor expansion. However, its specific effects on cell senescence within the context of OSCC have remained shrouded in uncertainty. This study delves into the mechanisms of EVO’s impact on OSCC by harnessing databases such as the Traditional Chinese Medicine Systems Pharmacology Database and Analysis Platform (TCMSP), The Cancer Genome Atlas (TCGA), the Gene Expression Omnibus (GEO), and CellAge to pinpoint potential targets and carry out in-depth bioinformatics analysis. The findings reveal that EVO can markedly enhance the expression of the androgen receptor (AR) in OSCC cells, inducing cellular senescence and thereby inhibiting tumor progression. Furthermore, the research indicates that AR expression is considerably lower in OSCC tissues than in normal tissues. This low expression of AR in tumor tissues is closely associated with advanced clinical stages and unfavorable prognoses in HNSCC patients. These discoveries open up new avenues for therapeutic strategies, and suggest that AR holds promise as a potential therapeutic target for OSCC, and EVO may amplify its antitumor effects by enhancing AR-mediated cellular senescence in the treatment of OSCC.

## 1. Introduction

Head and neck cancer is one of the most common malignant tumors; epidemiological studies show that HNSCC ranks eighth in the global incidence rate of malignant tumors and twelfth in mortality rate. By 2020, the number of new cases of HNSCC worldwide was approximately 840,000, and it is expected to rise to 1 million by 2030, posing a significant threat to human life and health [1]. Since most head and neck cancers originate from the mucosal epithelium of the oral cavity, pharynx, and larynx, over 90% of head and neck cancers are squamous cell carcinomas, with OSCC being the main type of HNSCC. OSCC has complex genetics and significant tumor heterogeneity, making its clinical treatment challenging [2]. Over the past nearly 30 years, traditional treatment methods such as surgery and chemoradiotherapy have shown significant progress in the treatment of OSCC. However, the high recurrence rate and long-term toxicity of chemoradiotherapy have not significantly improved the survival rate and quality of life for patients with OSCC [3]. Therefore, with the continuous development of genomics and the deepening of research on natural small molecule drugs, based on a precise understanding of patients’ genetic differences, the reasonable selection of therapeutic drugs and targeted treatment plans can significantly improve the clinical prognosis for patients with OSCC.

Evodiamine is a natural indole alkaloid found in the fruit of the plant *Evodia rutaecarpa*, and it is one of its main active ingredients [4]. It can exert various pharmacological effects in many diseases, including malignant tumors, inflammatory diseases, and autoimmune diseases [5,6,7]. Relevant studies have shown that evodiamine has a topoisomerase inhibitor effect and can effectively inhibit tumor activity [8]. In addition, evodiamine has been found to exert good anticancer effects by inhibiting cell proliferation, apoptosis [9], invasion, migration [10], metabolic reprogramming [11], and so forth. Therefore, developing and utilizing evodiamine through nanotechnology platforms and exploring its new anticancer mechanisms have become research hotspots in recent years.

As the fundamental structural and functional units of living organisms, cells are subject to senescence, a core biological principle and characteristic inherent to all life forms. Senescent cells undergo significant morphological changes and lose their replication ability. The expression of genes and proteins in cells, as well as the secretion levels of various factors, can also affect senescent cells, causing them to be in a state of decline [12]. Studies have shown that inducing tumor cell senescence can effectively inhibit the occurrence and development of malignant tumors [13,14]. Therefore, how to induce tumor cell senescence through drugs has become a potential means of treating malignant tumors. However, whether evodiamine can exert its anticancer effect by inducing tumor cell senescence warrants further investigation.

The androgen receptor (AR) gene, which encodes the AR protein, is situated on the X chromosome at the locus Xq11.2-q12. The AR gene contains seven introns and seven exons, and its mRNA has two isoforms. The AR is widely distributed in various tissues of the human body, mediating the effects of androgens and playing important biological functions in male and female reproduction, growth, and development [15]. In its inactive state, AR often binds to chaperones such as heat shock protein 70 (HSP70) and heat shock protein 90 (HSP90) in the cytoplasm, while the activated AR transfers to the nucleus and recognizes the androgen response elements (AREs) in DNA in a dimeric form, exerting corresponding biological functions, which are crucial for human growth, development, and health status [16,17]. Identified as a risk factor, AR exerts a deleterious influence in prostate cancer. Conversely, in liver cancer, AR can suppress the metastatic potential of hepatic cancer cells by modulating their migratory behavior and facilitating anoikis. Additionally, the AR can also induce tumor cell senescence by regulating the expression of P21 and tumor protein 63 (P63). While the abnormal expression and dysregulation of the AR are implicated in the occurrence and progression of malignancies such as prostate cancer, breast cancer, and liver cancer [18,19,20,21,22], its specific roles in HNSCC remain to be elucidated.

Against this backdrop, we conducted an in-depth analysis of the transcriptome data of HNSCC (including OSCC), using bioinformatics techniques based on databases such as TCGA and GEO. Simultaneously, we utilize OSCC as a prototypical representative of HNSCC and perform modern biological experiments based on human OSCC tissues and cell lines, such as Western blotting, to corroborate the findings derived from bioinformatics analyses. Our preliminary findings indicate that evodiamine may induce senescence in OSCC cells through the modulation of AR expression, consequently suppressing OSCC cell activity.

## 2. Materials and Methods

### 2.1. Materials

Evodiamine (Cat.HY-N0114) was provided by MedChemExpress (Shanghai, China), dissolved in dimethyl sulfoxide (DMSO) (Cat.D8371, Solarbio, Beijing, China), and stored at −20 °C. Primary antibodies of glyceraldehyde-3-phosphate dehydrogenase (GAPDH) (Cat.GB15002), AR (Cat.GB155311), cyclin-dependent kinase inhibitor 2A (CDKN2A/P16) (Cat.GB151602), cyclin-dependent kinase inhibitor 1A (CDKN1A/P21) (Cat.GB115313), tumor protein 53 (P53) (Cat.GB15627), horseradish peroxidase (HRP) conjugated goat anti-rabbit IgG (H+L) (Cat.GB23303), and HRP conjugated goat anti-mouse IgG (H+L) (Cat.GB23301) were provided by Servicebio (Wuhan, China). Protein Ladder M1 (Cat. SB-26619, 8–250 kDa) was provided by Sharebio (Shanghai, China), Protein Ladder M2 (Cat. G2083-250UL, 8–200 kDa) was provided by Servicebio (Wuhan, China). AR agonist YK11 (T7358) and inhibitor Ailanthone (AIL) (TQ0209) were provided by Topscience (Shanghai, China).

### 2.2. Cell Culture and Processing

The human OSCC cell lines (CAL27, SAS, and SCC9) were purchased from the American Type Culture Collection (Manassas, VA, USA). CAL27 cells were cultured in DMEM/high glucose (Cat.G4515-500ML, Servicebio, Wuhan, China), SAS cells were cultured in DMEM/F12 (Cat.G4612-500ML, Servicebio, Wuhan, China), and SCC9 cells were cultured in DMEM/F12 supplemented with 400 ng/mL hydrocortisone (Cat.A10188, Yuanye, Shanghai, China). All of them were supplemented with 10% fetal bovine serum and 1% penicillin–streptomycin solution (P/S) under standard conditions at 37 °C and 5% CO_2_.

### 2.3. Sample Collection

All OSCC tissues and adjacent non-cancerous tissues were obtained from OSCC patients who underwent surgical resection at the First Hospital of Lanzhou University between 2016 and 2023. After being fixed with paraformaldehyde, all OSCC tissues and paracancer tissues were embedded in paraffin and stored at room temperature. The OSCC patients included in this study were all diagnosed with primary OSCC, excluding other types of malignant tumors. This study was approved by the Ethics Committee of the First Hospital of Lanzhou University (No. LDYYLL2024-776, date: 19 December 2024). The exemption of informed consent in this study is based on the following two criteria: ① vulnerable populations under special circumstances (e.g., patients may have significant difficulties in understanding informed consent due to low educational levels or lack of knowledge about medical information. If the study offers substantial health benefits to the local patient population and the risks are manageable, and after thorough consideration of ethical factors and approval by the ethics committee, exemption from informed consent may be granted) and ② strict review by the ethics committee (the ethics committee comprehensively considers multiple factors, including the purpose of the study, risks and benefits, and special circumstances of patients, to ensure that the decision to exempt informed consent is based on sufficient justification and adequate protection of patient rights. The ethics committee approves the exemption only if it believes that the exemption will not harm patients’ legitimate rights and interests and that the conduct of the study complies with ethical principles and legal requirements). These criteria align with the standards for exempting informed consent for certain patients in clinical research. Based on these criteria, we meet with patients and their families before surgery to inform them about the use of biological samples in scientific research and obtain their consent.

### 2.4. Data Download and Analysis Using the Public Database

Using the TCGAbiolinks package in R software (v4.0.3), HNSCC RNA-Seq data (including 504 HNSCC tissues and 44 normal tissues) were downloaded from the TCGA database. Clinical information such as age, gender, and pathological stage were sourced from TCGA-HNSCC. The RNA-Seq data underwent differential analysis using the edgeR package. The GSE138206 and GSE172577 datasets were sourced from the GEO database (https://www.ncbi.nlm.nih.gov/geo/, accessed on 6 November 2024). The significance threshold was set as |log_2_(fold change [FC])| > 1 and *p* < 0.05.

### 2.5. Target Screening in TCMSP Database

Traditional Chinese Medicine Systems Pharmacology (https://www.tcmsp-e.com/#/database, accessed on 10 November 2024) was used to retrieve and query the targets, associated diseases, and related pharmacokinetic data of traditional Chinese medicines or natural small molecule compounds. Utilizing the TCMSP database, with evodiamine as the keyword, the target data of evodiamine were retrieved and downloaded, and EVO target screening was conducted.

### 2.6. CellAge Database Analysis

The CellAge database (https://genomics.senescence.info/cells/, accessed on 12 November 2024) was used to analyze the correlation between differential genes and cellular senescence. Utilizing the CellAge database, we conducted a cellular senescence correlation analysis on differentially expressed genes related to HNSCC in the TCGA and GEO databases.

### 2.7. Pan-Cancer Correlation Analysis

The Human Protein Atlas (HPA) database (https://www.proteinatlas.org/, accessed on 13 November 2024) was used to verify AR expression in various normal human tissues. The University of California Santa Cruz (UCSC) database (https://genome-asia.ucsc.edu/index.html, accessed on 13 November 2024) was consulted to explore AR gene annotation information. Genetic alterations of AR across different tumors in the TCGA database were investigated using the cBioPortal database (https://www.cbioportal.org/, accessed on 13 November 2024).

Utilizing the Gene Expression Profiling Interactive Analysis (GEPIA) database (http://gepia.cancer-pku.cn/, accessed on 14 November 2024), we obtained the expression transcriptome of AR in pan-cancer. Based on the tumor and normal data in the TCGA database, as well as the normal data in the The Genotype-Tissue Expression (GETx) database, we constructed box plots for AR expression, with a log_2_FC threshold set at 1 and a *p*-value set at 0.01.

### 2.8. Survival Analysis

TIMER 2.0 (http://timer.cistrome.org/, accessed on 15 November 2024) and Kaplan–Meier Plotter (https://kmplot.com/analysis/index.php?p=service&cancer=pancancer_rnaseq, accessed on 15 November 2024) databases were used to explore the correlation between genes and the prognosis of different tumors. Through these two databases, we explored the prognostic value of AR in overall survival rates in pan-cancer and HNSCC.

### 2.9. Expression Analysis of the AR in Cell Lines in the High-Throughput Omics-Based Tumor Roadmap on the Shiny (ShinyTHOR) App

The ShinyTHOR App (https://alexismurillo.shinyapps.io/ShinyThor/, accessed on 28 June 2025) is a free, open-access web application designed for laboratory researchers to intuitively access multi-omics and drug-related data [23]. It integrates datasets from the Cancer Cell Line Encyclopedia (CCLE), miRTarBase, circInteractome, and cancer drug sensitivity genomics. ShinyTHOR enables users to assess the associations between selected cancer cell line omics analytes, drug responses, and gene characteristics, thereby simplifying data integration and analysis. The application, built using R and HTML via the Shiny app, combines CCLE data visualization with an intuitive interface, streamlining experimental planning in cancer research. We utilized this app to compare the expression levels of AR in upper aerodigestive tract cell lines.

### 2.10. Immunohistochemistry (IHC)

Immunohistochemical staining was performed on cancerous and adjacent non-cancerous tissue sections from 10 patients with OSCC. Following standard procedures, the tissues were stained with AR primary antibody (1:1500, Servicebio, Wuhan, China). The semiquantitative analysis for IHC results was made by Image J software (v1.53) [24].

### 2.11. Cell Counting Kit-8 (CCK8) Assay

The CCK-8 detection was conducted using the CCK-8 Cell Proliferation and Cytotoxicity Assay Kit (Cat.CA1210, Solarbio, Beijing, China). According to the instructions, the CCK-8 solution was added to the sample wells (3–6 replicates for each sample), incubated at 37 °C in a 5% CO_2_ environment for 2 h, and then the absorbance at 450 nm was measured using a microplate reader(Thermo Fisher Scientific, Waltham, MA, USA).

### 2.12. Cell Cycle and Apoptosis Examination

CAL27, SAS, and SCC9 cells (2 × 10^6^ cells) were collected for testing, washed three times with cold phosphate-buffered saline (PBS), and fixed overnight with 70% ethanol. The cells were resuspended with 100 μL Ribonuclease A (RNase A), incubated in a water bath at 37 °C for 30 min, supplemented with 400 μL Propidium (PI, 50 μg/mL) staining solution, incubated in the dark at 4 °C for 30 min, and finally analyzed by flow cytometry (BD Biosciences, Franklin Lake, NJ, USA).

CAL27, SAS, and SCC9 cells and their culture medium supernatant were collected for detection. After centrifuging at 1000 rpm for 7 min, and they were washed twice with pre-cooled PBS. The cells were resuspended with 250 μL of diluted binding buffer, adjusting the concentration to 1 × 10^6^/mL. A total of 100 μL of the cell suspension was transferred into a 5 mL flow tube. A total of 5 μL of Annexin V-FITC was added and gently mixed. The suspension was then incubated at room temperature (20–25 °C), in the dark, for 10 min. Five minutes before running the instrument, 10 μL of propidium iodide solution were added and gently mixed. Before running the instrument, 400 μL of PBS were added to the reaction tube to resuspend the cells. They were stored in the dark and flow cytometry detection was performed immediately (BD Biosciences, Franklin Lake, NJ, USA).

### 2.13. Cell Colony Formation

CAL27 cells were seeded into a 6-well plate (1 × 10^3^ cells/well) and cultured for 14 days. The cells were fixed in 4% paraformaldehyde solution for 30 min, stained with 1% crystal violet for 15 min, and then the colonies were counted.

### 2.14. 5-Ethynyl-2’-deoxyuridine (EdU) Detection

An appropriate number of cells were cultured in a 6-well plate (cover slips were added if necessary). The cells were labelled with pre-warmed EdU working solution at a final concentration of 10 μM (continue incubating for 2 h). After labeling, the cells were fixed with fixing solution at room temperature for 15 minutes and washed the 3 times (3–5 min per wash). After fixation, the cells were incubated with permeabilization solution at room temperature for 10–15 min to permeabilize the cells. After permeabilization, the cells were washed 1–2 times (3–5 min per wash). After washing, Click reaction solution was added and the cells were incubated at room temperature in the dark for 30 min, followed by washing the cells 3 times (3–5 min per wash). Then, Hoechst 33342 (1×) (dilute Hoechst 33342 (1000×) with PBS at a 1:1000 ratio) was added and cells were incubated at room temperature in the dark for 10 min. After washing the cells 3 times (3–5 min per wash), they were observed under a fluorescence microscope. The above experimental steps and reagent formulas were carried out according to the instructions of the BeyoClick™ EdU-488 Cell Proliferation Assay Kit (Catalog No. C0071S, Beyotime, Beijing, China).

### 2.15. β-Galactosidase Detection

The β-galactosidase detection was conducted using the Senescence-Associated β-Galactosidase (SA-β-Gal) Stain Kit (Cat.G1580, Solarbio, Beijing, China), following the manufacturer’s instructions.

### 2.16. Quantitative Reverse Transcription Polymerase Chain Reaction (RT-qPCR)

Total RNA was extracted using the M5 HiPer Universal RNA Mini Kit (Cat. MF036-01, Mei5bio, Beijing, China), and then reverse transcribed using NovoScript^®^Plus All-in-one 1st Strand cDNA Synthesis SuperMix (gDNA Purge) (Cat. E047, Novoprotein, Suzhou, China). qPCR was performed using the Hieff^®^ qPCR SYBR Green Master Mix (Low Rox Plus) (Cat. 11202ES08, Yeasen, Shanghai, China). Human GAPDH was used as an internal control, and the relative expression level of mRNA was calculated using the 2^−ΔΔCt^ method and normalized to the internal control [11]. After retrieving the coding sequence (CDS) for the AR (Gene ID: 367), GAPDH (Gene ID: 2597), P21 (Gene ID: 1026), and P53 (Gene ID: 7157) from the National Center for Biotechnology Information (NCBI) database, they were submitted to Sangon Biotech (Shanghai, China) for primer design. The primer for P16 was derived from a reference [25], and all primers were synthesized by Tsingke Biotech (Beijing, China). The specific sequences of the primers used for qRT-PCR are available in Table 1.The melting curve is shown in Figure S7.

### 2.17. Protein Extraction, Protein Concentration Measurement, and Western Blotting

Fully digest the cells in the well plate with trypsin, and then lyse the cells thoroughly with radio immunoprecipitation assay (RIPA) lysis buffer containing phenylmethanesulfonyl fluoride (PMSF) on ice. Centrifuge the lysate at high speed (4 °C, 14,000× *g*, 15 min) to separate the supernatant. Use the bicinchoninic acid (BCA) assay kit (Cat.PC0020, Solarbio, Beijing, China) to determine the protein concentration. Subsequently, use the sodium dodecyl sulfate- polyacrylamide gel electrophoresis (SDS-PAGE) gel kit (Cat.P1200, Solarbio, Beijing, China) for SDS-PAGE gel electrophoresis. After completion, transfer the protein to a hydrophobic polyvinylidene fluoride (PVDF) membrane (Cat.IPVH00010, Merck-Millipore, Billerica, MA, USA). Use GAPDH as an internal standard. Detect the protein bands with an enhanced chemiluminescence (ECL) reagent (Cat.WBKLS0100, Merck-Millipore, Billerica, MA, USA). The primary antibodies used in Western blotting, GAPDH, P16, and P53, were monoclonal antibodies, and P21 was a polyclonal antibody. Among these, GAPDH and P16 were mouse-derived antibodies, and P21 and P53 were rabbit-derived antibodies. The concentrations of the primary antibodies were as follows: GAPDH (1:5000), P16 (1:1000), P21 (1:600), and P53 (1:1000).

### 2.18. Statistics and Analysis

Data analysis was performed using Statistical Package for the Social Sciences (SPSS) 25.0 software and GraphPad Prism 9.0. Kaplan–Meier curves and log-rank tests were employed to evaluate progression-free survival (PFS) and overall survival (OS) outcomes. Differences between two groups were assessed using Student’s *t*-test. A *p*-value < 0.05 was considered statistically significant.

## 3. Results

### 3.1. Screening of Differential Genes and Selection of Key Genes in HNSCC

**Figure 1:**
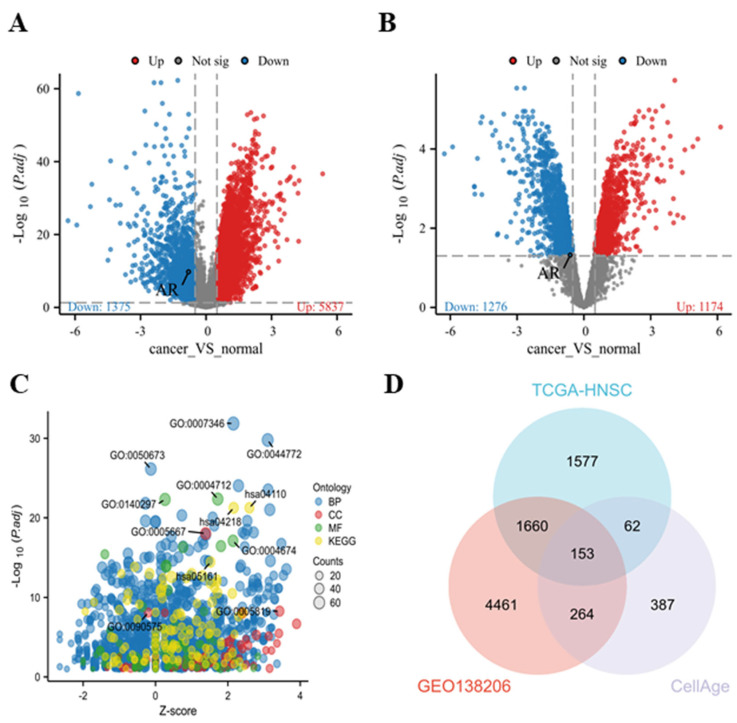
Differential expression landscape of genes in HNSCC. (**A**) Volcano plot of gene expression in HNSCC tissues and normal tissues in TCGA-HNSCC. Red and blue data points represent upregulated and downregulated genes, respectively. (**B**) Volcano plot of gene expression in OSCC tissues and normal tissues in GEO-GSE138206. Red and blue data points represent upregulated and downregulated genes, respectively. (**C**) Bubble chart of GO and KEGG enrichment analysis of differentially expressed genes. Blue, red, green, and yellow dots represent GO-BP, GO-CC, GO-MF, and KEGG pathways, respectively. (**D**) Venn diagram of TCGA-HNSCC dataset, GEO-GSE138206 dataset, and CellAge database. The Benjamini and Hochberg (BH) method was used to adjust *p*-values of the data in (**A**,**B**).

To screen for differentially expressed genes (DEGs) related to HNSCC, we downloaded the TCGA-HNSC transcriptome data of 504 HNSCC tissues and 44 normal tissues from the TCGA database, and the GSE138206 HNSCC transcriptome dataset from the GEO database. We then analyzed these datasets. The volcano plots displayed the upregulated (red) and downregulated (green) DEGs in the TCGA-HNSC and GSE138206 datasets (Figure 1A,B). Through Gene Ontology (GO) and Kyoto Encyclopedia of Genes and Genomes (KEGG) enrichment analyses, we observed a significant enrichment of cellular senescence (hsa04218) within the KEGG pathways, hinting at a potential close association between cellular senescence and the onset and progression of HNSCC (Figure 1C, Table S1). Therefore, based on the cell senescence-related gene dataset in the CellAge database, we overlapped the above two sets of DEGs with the cell senescence dataset in the CellAge database, obtaining a total of 153 DEGs (Figure 1D). Finally, we combined with the related targets of EVO in the TCSMP database (Table 2). We concluded that the AR, as a target of EVO, may be closely related to HNSCC and cell senescence.

### 3.2. Tissue-Specific Expression of AR in Pan-Cancers

**Figure 2:**
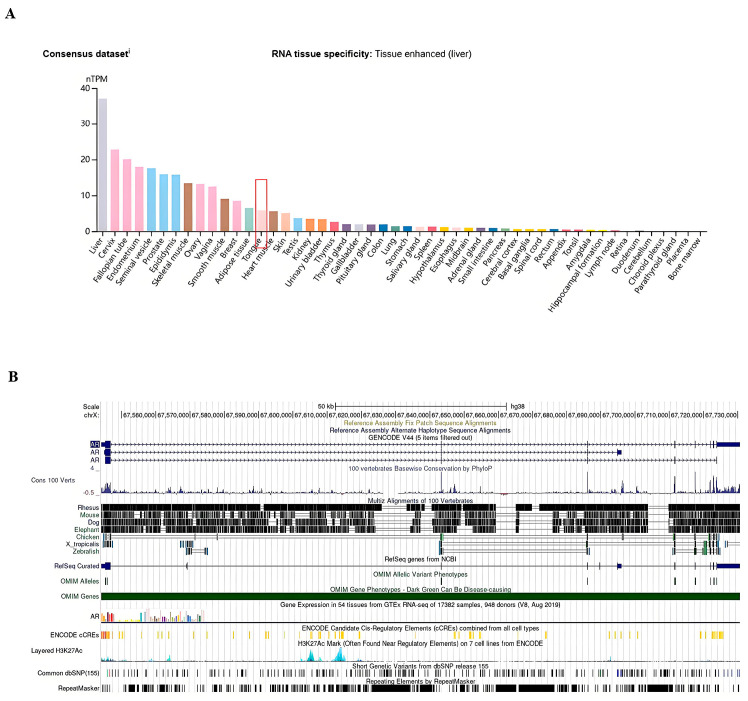

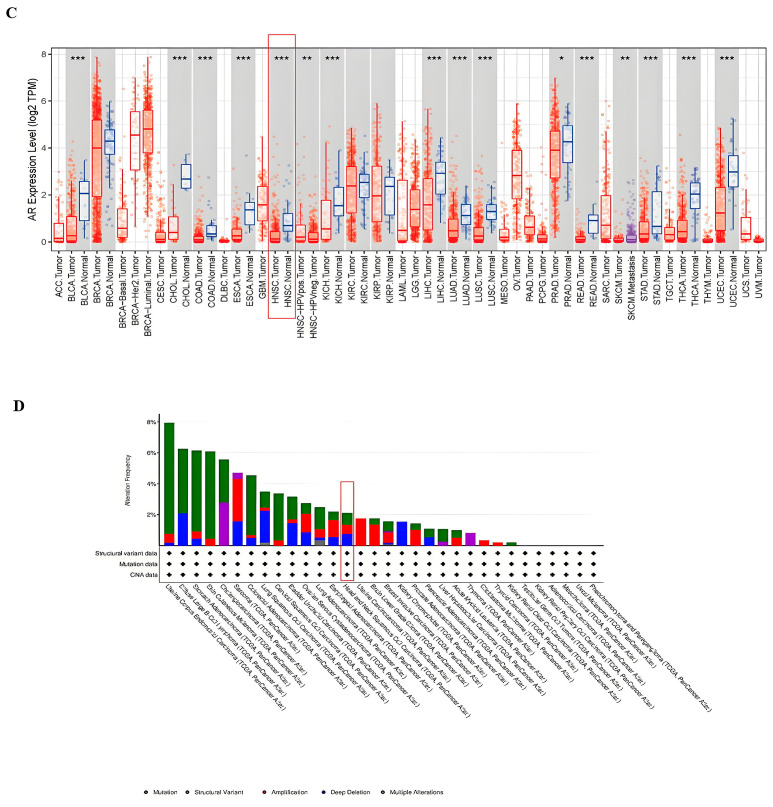
Differential expression of AR in normal and various tumor tissues as well as its evolutionary conservation in vertebrates. (**A**) Analysis of AR expression in normal tissues based on the HPA database and GTEx and FANTOM5 (Functional Annotation of Mammalian Genomes) datasets. (**B**) Analysis of AR conservation in vertebrates using the UCSC genome browser. (**C**) Visualization of AR expression in different tumor types through TIMER 2.0. (**D**) Visualization of AR alterations in different tumors in the TCGA database using the cBioProtal database. The data highlighted in red frame represents the data of patients with head and neck cancer. * *p* < 0.05; ** *p* < 0.01; and *** *p* < 0.001.

We validated the expression of the AR in various normal tissues of the human body through the HPA, GTEx, and Functional Annotation of The Mammalian Genome 5 (FANTOM5) datasets in the HPA database. The results showed that the AR is highly expressed in the tongue of the head and neck region (Figure 2A). Additionally, through the UCSC database, we also discovered that the AR is relatively conserved in vertebrates (Figure 2B). By studying the expression level of the AR in tumor tissues, we found that the AR is expressed to some extent in various types of tumors and related distant metastatic tumors. Specifically, the expression level of AR mRNA in tumor tissues of head and neck squamous cell carcinoma (HNSC), bladder urothelial carcinoma (BLCA), cholangiocarcinoma (CHOL), colon adenocarcinoma (COAD), esophageal cancer (ESCA), renal chromophobe carcinoma (KICH), renal clear cell carcinoma (KIRC), liver hepatocellular carcinoma (LIHC), lung adenocarcinoma (LUAD), lung squamous cell carcinoma (LUSC), prostate adenocarcinoma (PRAD), rectal adenocarcinoma (READ), skin cutaneous melanoma (SKCM), gastric adenocarcinoma (STAD), thyroid carcinoma (THCA), and uterine corpus endometrial carcinoma (UCEC) is significantly lower than that in corresponding normal tissues or distant metastatic tumors (Figure 2C). We also conducted a study on the genetic alterations of the AR in different tumors in the TCGA database through the cBioPortal database. The results showed that the genetic alterations of the AR in HNSCC tumor samples include copy number deletions, point mutations, and copy number amplifications, with copy number deletions and point mutations accounting for the majority (Figure 2D).

### 3.3. Downregulated Expression of AR Is Associated with the Survival Rate of HNSCC Patients

**Figure 3:**
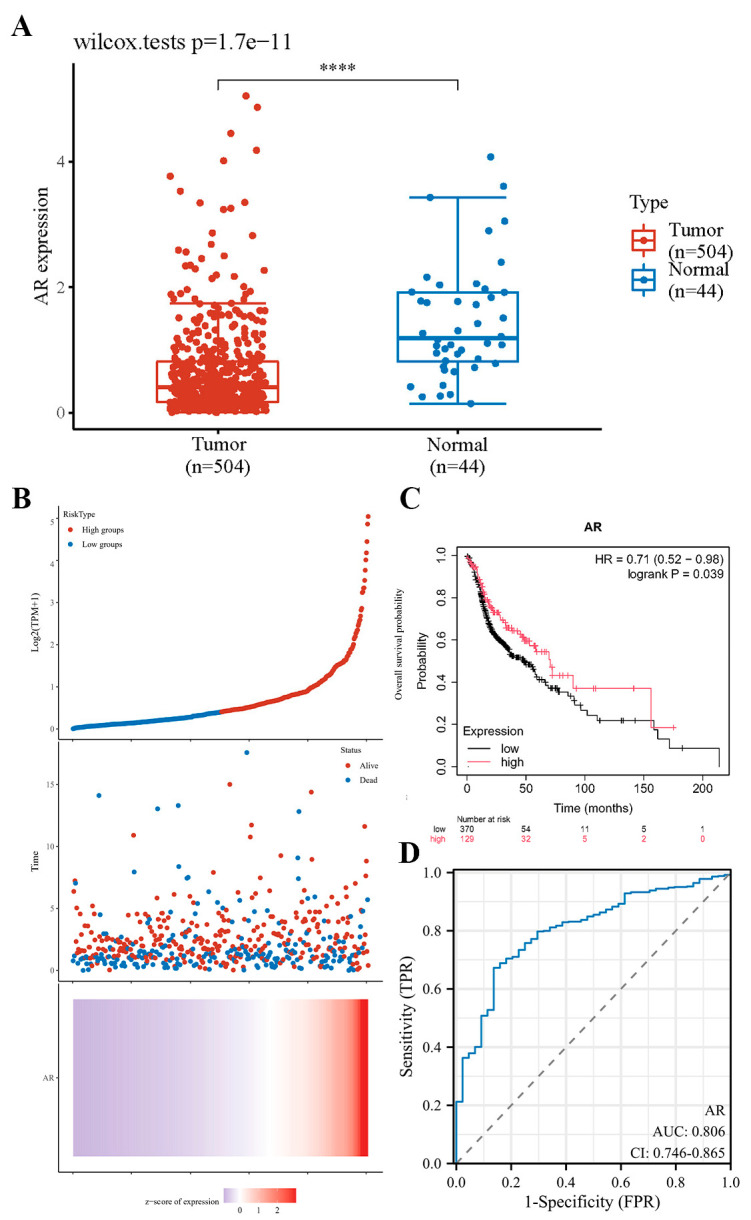
Downregulated AR expression is associated with poor prognosis in HNSCC. (**A**) In the TCGA database, AR expression levels are significantly downregulated in HNSCC. (**B**) The risk score for each HNSCC patient increases from blue to red, representing the survival time and AR-related characteristics of each HNSCC patient. (**C**) In HNSCC patients, downregulation of AR expression is associated with poor prognosis. (**D**) ROC curve for AR-related characteristic scores. **** *p* < 0.0001.

Based on the TCGA database, the significant expression of the AR in HNSCC tissues was once again verified (Figure 3A). Furthermore, we categorized each HNSCC patient in the database into high and low AR expression groups based on the median AR expression level derived from TCGA data (Figure 3B). Subsequently, we used the Kaplan–Meier Plotter database to plot survival curves of HNSCC patients and found that a decrease in AR expression level was associated with poor overall survival (OS) in HNSCC patients (Figure 3C). Furthermore, through the plotting of the receiver operating characteristic (ROC) curve, we have also discovered that the AR holds a degree of significance in the diagnosis of HNSCC (Figure 3D).

### 3.4. Correlational Analysis of AR Expression Aberrations with Clinical Characteristics in HNSCC

**Figure 4:**
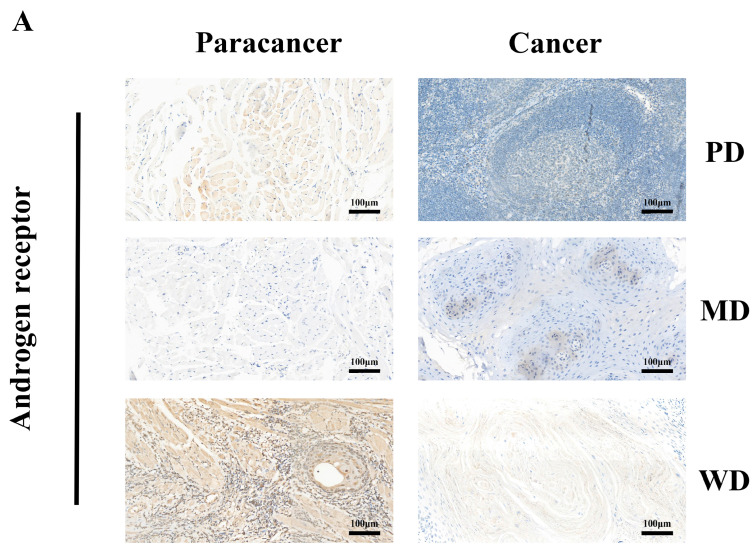

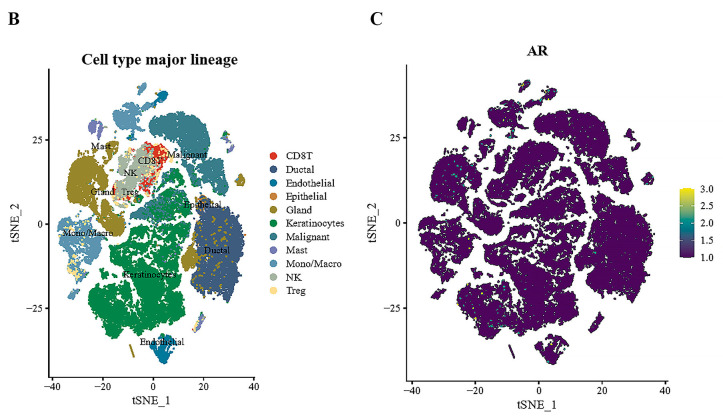
AR expression profiles in tissues and different cell subpopulations of OSCC and adjacent non-cancerous tissues by immunohistochemical detection. (**A**) AR IHC staining in OSCC tissues of varying differentiation levels and adjacent non-cancerous tissues. (**B**) Different cell subpopulations in OSCC tissues. (**C**) Expression levels of AR in different cell subpopulations of OSCC tissues.

**Figure 5:**
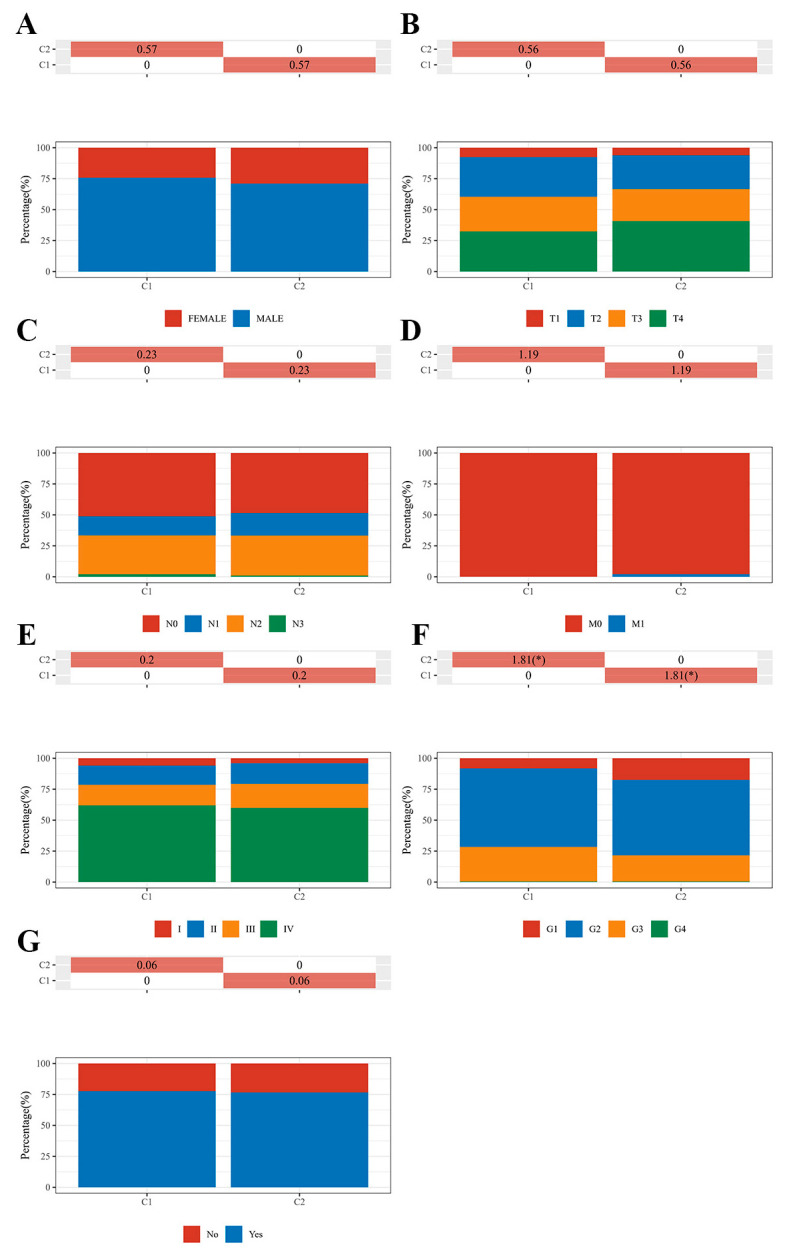
Comparison of clinical information for patients with different expression levels of AR in the TCGA-HNSCC dataset. (**A**) Downregulation of AR is not associated with gender. (**B**) Downregulation of AR is not associated with lymph node metastasis. (**C**) Downregulation of AR is not associated with distant metastasis. (**D**) Downregulation of AR is not associated with poor T stage. (**E**) Downregulation of AR is not associated with poor pathological stage. (**F**) Downregulation of AR is associated with poor histopathological grade. (**G**) Downregulation of AR is not associated with smoking history. * *p* < 0.05.

To verify the results of the Kaplan–Meier survival curve drawn based on the TCGA database, we collected cancerous and adjacent tissues from 10 OSCC patients with varying degrees of differentiation for immunohistochemical staining (Table S2). We found that the expression of the AR in adjacent tissues was significantly higher than that in cancerous tissues, which is consistent with the Kaplan–Meier survival curve results of HNSCC patients in the TCGA database. Additionally, through the analysis of IHC, we found that the expression of the AR in the para-cancer tissues of OSCC patients was significantly higher than in the cancer tissues (Figure 4A), and the expression of the AR in the cancer tissues of well-differentiated patients was significantly higher than that in poorly differentiated patients (*p* < 0.01) (Figure S1). This suggests that the expression of AR is correlated with the degree of differentiation of cancer tissues, with higher expression levels corresponding to higher levels of differentiation. Meanwhile, based on the GEO database GSE172577 dataset, we also conducted a single-cell analysis of OSCC. We found that AR expression levels were similarly low in different cell subpopulations of OSCC tissues (Figure 4B,C). To validate the effectiveness of the Kaplan–Meier curve, we also conducted a statistical analysis on the clinical baseline data for 504 samples in the TCGA-HNSCC dataset. Based on the TCGA-HNSCC database, we analyzed the distribution of clinical information including gender, TNM stage, grade, and smoking history between with patients high AR and low AR expression (Figure 5A–G). The results indicate that the expression level of the AR is significantly correlated with the pathological classification of HNSCC patients (Figure 5F). Therefore, we preliminarily conclude that an abnormal expression of the AR plays a significant role in the occurrence and development of HNSCC.

### 3.5. Evodiamine Suppresses OSCC Cell Proliferation In Vitro and Induces G2/M Arrest and Apoptosis

**Figure 6:**
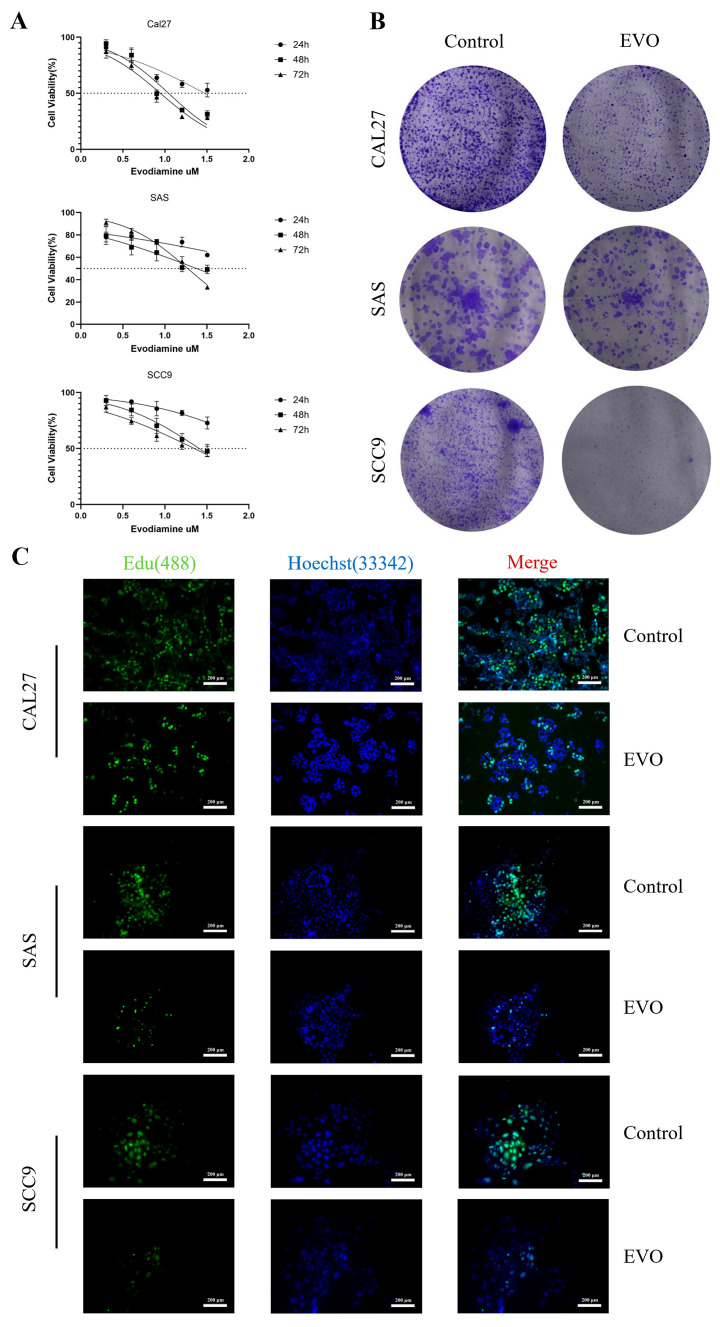

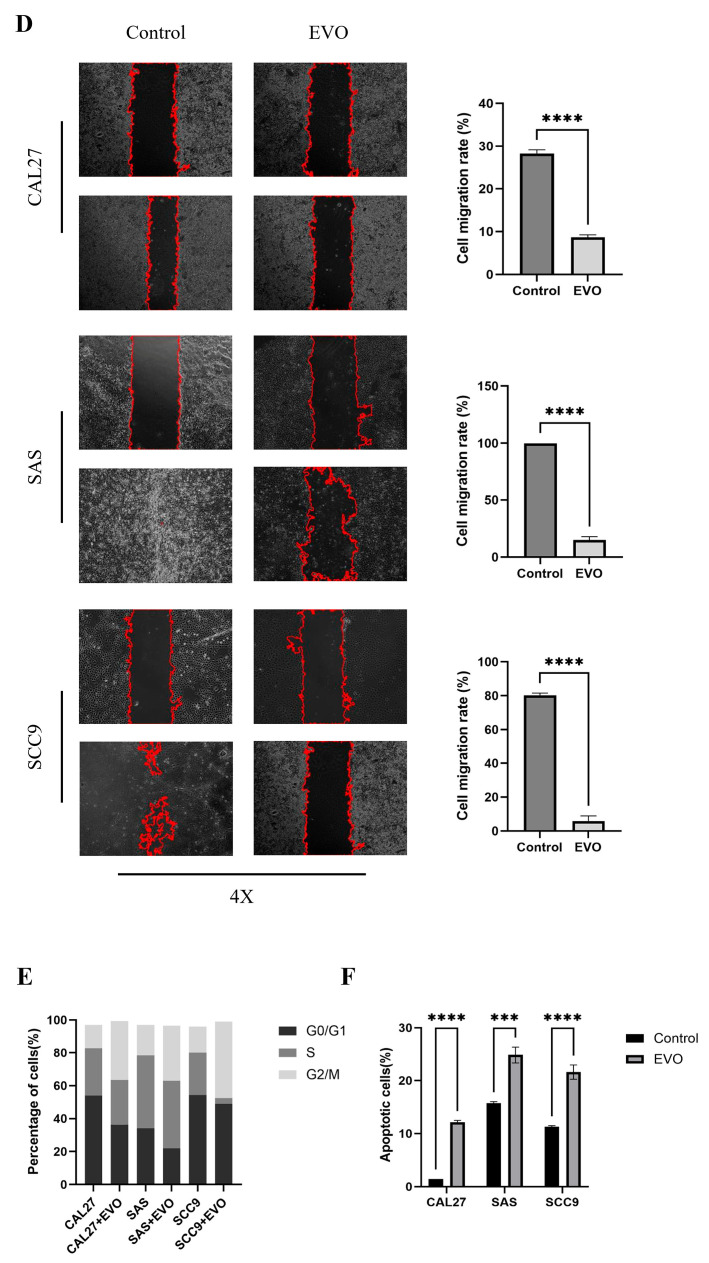
Evodiamine inhibits proliferation of human OSCC cells and induces apoptosis in vitro. (**A**) The optimal concentration of EVO for intervening in CAL27, SAS, and SCC9 cells was determined through the CCK8 assay. (**B**) The colony formation of OSCC cells was assessed under both EVO-treated and untreated conditions, revealing that EVO can inhibit the formation of OSCC cell colonies. (**C**) The effect of EVO on the proliferative capacity of OSCC cells was detected using the EdU method, indicating that EVO can suppress the proliferative ability of OSCC cells. (**D**) Wound healing experiments evaluated the migration ability of OSCC cells under both EVO-treated and untreated conditions, demonstrating that EVO effectively inhibits the migration ability of OSCC cells. (**E**,**F**) Flow cytometry was used to detect cell apoptosis after 48 h of EVO treatment, revealing that EVO can induce G2/M phase arrest and apoptosis in OSCC cells. *** *p* < 0.001, **** *p* < 0.0001.

To verify the therapeutic effect of evodiamine on OSCC, we selected CAL27, SAS, and SCC9 cells as subjects for intervention. Through CCK8 experiments, we screened the optimal concentration of EVO for subsequent experiments and evaluated its impact on the proliferation ability of OSCC cells (EVO’s inhibitory effect increases with increasing concentration and time). Finally, we selected 11.08 μM, 23.98 μM, and 25.65 μM as the optimal concentrations of EVO for a 48 h intervention in CAL27, SAS, and SCC9 cells, respectively, for subsequent experiments (Figure 6A). Cell colony formation experiments and EdU experiments were conducted to evaluate the impact of EVO on the proliferation ability of CAL27, SAS, and SCC9 cells. We found that EVO had a certain inhibitory effect on the proliferation ability of CAL27, SAS, and SCC9 cells, with the most significant inhibition observed in CAL27 cells (Figure 6B,C). Wound healing experiments were conducted to evaluate the impact of EVO on the migration ability of CAL27, SAS, and SCC9 cells. Compared with the control group, EVO had an inhibitory effect on the migration ability of CAL27, SAS, and SCC9 cells (Figure 6D). In addition, flow cytometry was used to detect the impact of EVO on the cell cycle and apoptosis. Compared with the control group, CAL27, SAS, and SCC9 cells after EVO intervention showed significant G2/M arrest in the cell cycle, and the proportion of apoptotic cells increased significantly (Figure 6E,F) and (Figures S2 and S3).

### 3.6. Stimulated AR Overexpression in OSCC Cells Promotes Senescence and Halts Cell Activities

**Figure 7:**
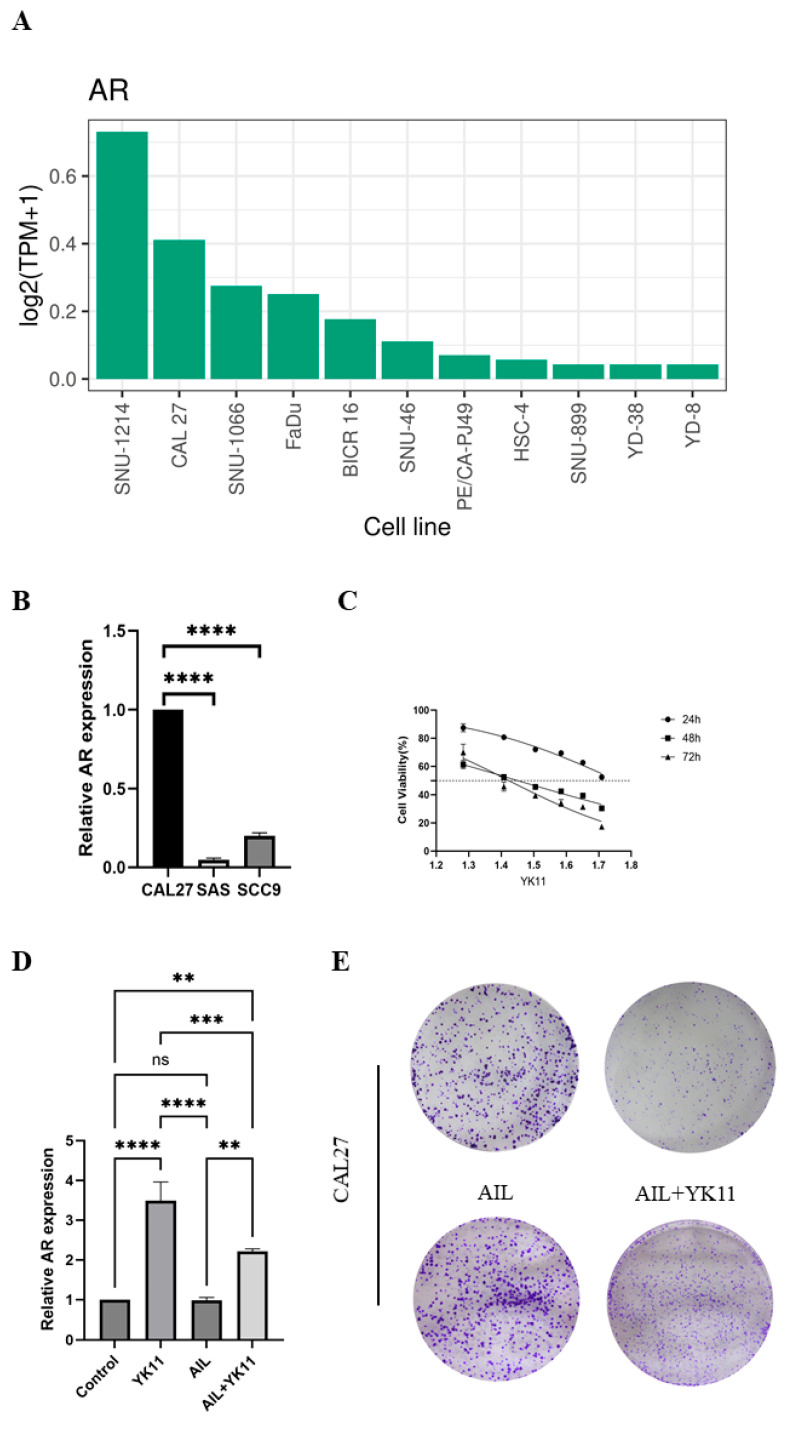

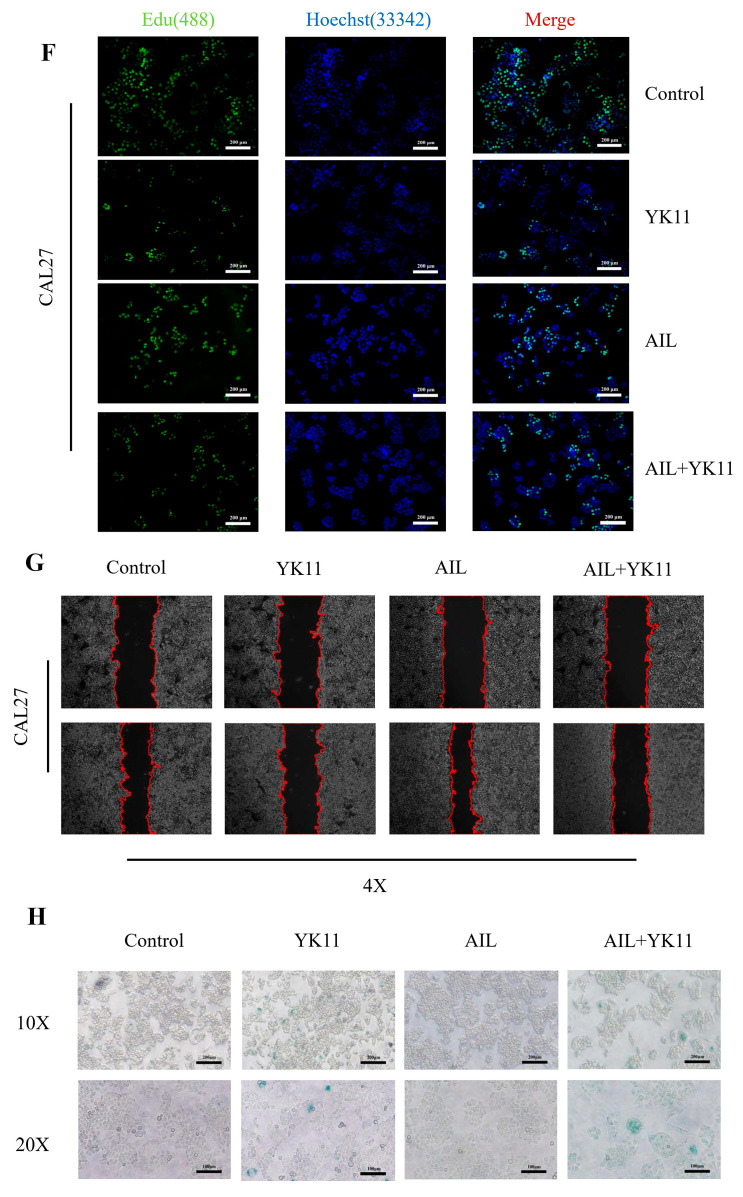

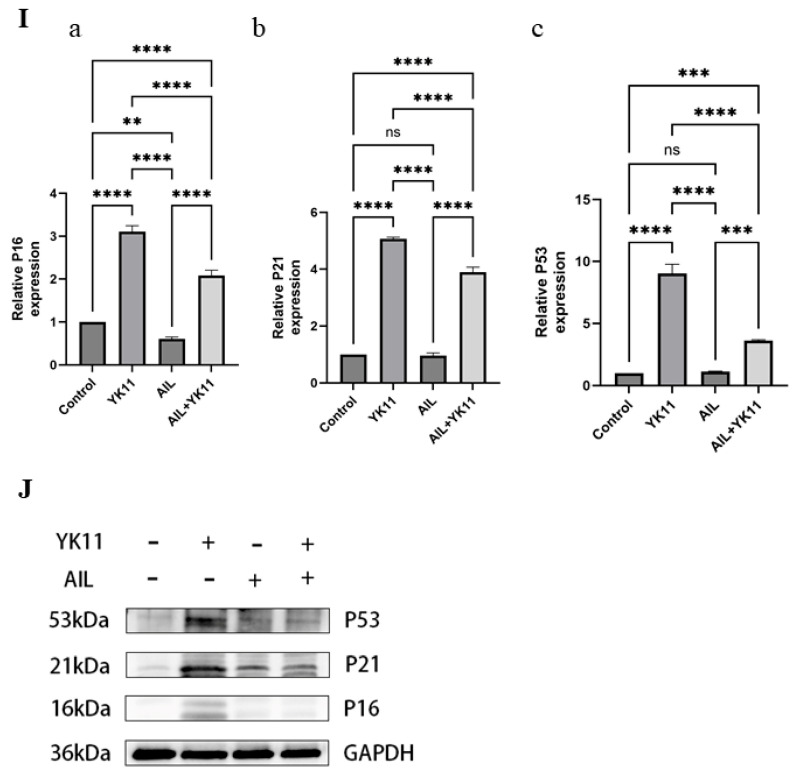
Expression of AR in different OSCC cells and the impact of overexpression on cellular senescence and activities. (**A**) AR expression levels in upper aerodigestive tract cell lines. (**B**) AR expression levels in human OSCC cell lines CAL27, SAS, and SCC9. (**C**) Determination of the optimal concentration of AR agonist YK11 for intervening in CAL27 cells through the CCK8 assay. (**D**) Measurement of AR expression levels in CAL27 cells with and without intervention by YK11, AIL, and AIL+YK11. YK11 significantly increased the expression level of AR. (**E**) The effect of YK11, AIL, and AIL+YK11 intervention on colony formation in CAL27 cells. YK11-induced AR overexpression effectively inhibited the formation of CAL27 cell colonies. (**F**) Detection of the effect of AR overexpression on the proliferative capacity of CAL27 cells using the EdU method. YK11-induced AR overexpression inhibited the proliferative capacity of OSCC cells. (**G**) Wound healing assay to evaluate the effect of AR overexpression on the migration ability of CAL27 cells. YK11-induced AR overexpression effectively inhibited the migration ability of OSCC cells. (**H**) Measurement of cell senescence using the SA-β-Gal assay. Blue-green color indicates senescent cells. The number of senescent CAL27 cells significantly increased after AR overexpression. (**I**) (a–c) Expression levels of p16, p21, and p53 mRNA in CAL27 cells in each group, after intervention with YK11 and AIL. (**J**) Expression levels of senescence-related proteins. ns *p* > 0.05, ** *p* < 0.01, and *** *p* < 0.001, **** *p* < 0.0001. The unprocessed Western blot image is shown in Figure S6.

The ShinyTHOR App indicates that the AR expression level in CAL27 cells is significantly higher than that in SAS and SCC9 cells in upper aerodigestive tract cell lines (Figure 7A, Table S3). We employed qPCR to assess the expression levels of AR mRNA in the three cell lines. The qPCR results revealed that CAL27 had the highest AR mRNA expression level among the three cell lines, aligning with the data from the ShinyTHOR App (Figure 7B). Based on this, we selected CAL27 cells for subsequent experiments. To further investigate the effects of AR on OSCC cells, we divided the cells into three groups and simultaneously used AR agonist YK11, AR antagonist AIL, and AIL+YK11 to intervene in CAL27 cells, inducing/inhibiting their AR expression. Through the CCK8 assay, we determined that 25.6 μM was the optimal concentration for YK11 intervention in CAL27 cells. Additionally, since AIL effectively inhibits AR expression at 6.9 nM, we identified 6.9 nM as the optimal concentration for AIL intervention in CAL27 cells. Subsequently, we used YK11 and AIL at the aforementioned concentrations to intervene in CAL27 cells and conducted further experiments (Figure 7C). The results showed that the AR mRNA expression levels in CAL27 cells were significantly increased after YK11 and AIL+YK11 interventions compared to the control and AIL groups (Figure 7D). Colony formation assays and EdU assays were used to evaluate the effects of AR overexpression or under expression on the proliferation capacity of CAL27 cells. Compared to the control and AIL groups, the proliferation capacity of cells in the YK11 and AIL+YK11 groups was significantly reduced (Figure 7E,F). Wound healing assays were conducted to assess the effects of AR overexpression/under expression on the migration capacity of CAL27 cells. Compared to the control and AIL groups, the migration capacity of CAL27 cells in the YK11 and AIL+YK11 groups was significantly reduced (Figure 7G) and (Figure S4). Subsequently, we utilized SA-β-Gal staining to ascertain whether AR overexpression induced senescence in CAL27 cells. When compared to the control and AIL groups, cells in the YK11 and AIL+YK11 groups exhibited pronounced blue staining, indicative of heightened β-Gal accumulation and marked cellular senescence (Figure 7H). qPCR was used to detect the mRNA expression levels of P53, P16, and P21 before and after YK11 and AIL interventions. The results showed that after YK11 and AIL intervention, the expression levels of p16, p21, and p53 mRNA in CAL27 cells of the YK11 group were significantly higher than those in the control group, AIL group, and AIL+YK11 group (*p* < 0.0001). The expression levels of p16, p21, and p53 mRNA in CAL27 cells of the AIL+YK11 group were significantly higher than those in the control group and AIL group (*p* < 0.001, *p* < 0.0001). The expression level of p16 mRNA in CAL27 cells of the control group was higher than that in the AIL group (*p* < 0.01), but there was no significant difference in the expression levels of p21 and p53 mRNA (*p* > 0.05) (Figure 7I(a–c)). Western blotting was used to detect the protein expression levels of P53, P16, and P21 before and after YK11 and AIL interventions. The results showed that the protein levels of P53, P16, and P21 in CAL27 cells were significantly increased after YK11 and AIL+YK11 interventions (Figure 7J).

### 3.7. Evodiamine Triggers Senescence of OSCC Cells by Enhancing Expression of AR

**Figure 8:**
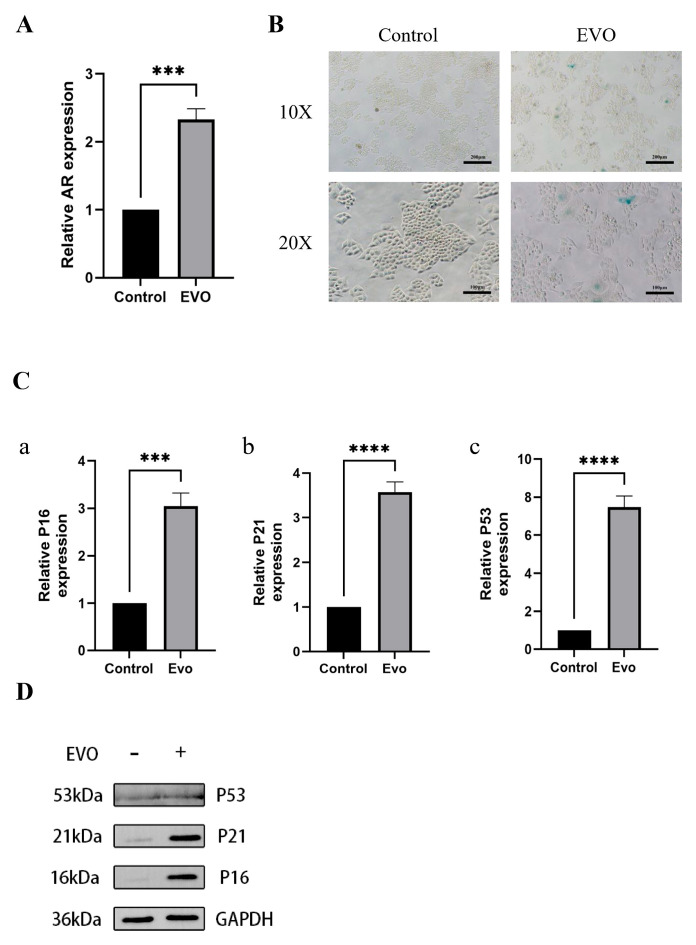
EVO induces OSCC cell senescence by upregulating AR expression. (**A**) EVO treatment of OSCC cells significantly increases the expression level of AR mRNA. (**B**) SA-β-Gal assay is used to determine the senescence status of OSCC cells after EVO treatment, with blue-green indicating senescent cells. (**C**) Expression levels of p16, p21, and p53 mRNA in CAL27 cells in each group, after intervention with EVO. (**D**) AR overexpression alters the expression levels of senescence-related proteins. Comparisons for WB between samples were derived from the same experiment and blots were processed in parallel. *** *p* < 0.001, **** *p* < 0.0001.

To further investigate the mechanism by which EVO and the AR affect OSCC cells, we first observed the effect of EVO on the expression level of AR in OSCC cells. We used RT-qPCR to detect the expression level of AR mRNA in OSCC cells before and after EVO intervention. The RT-qPCR results showed that compared with the control group, the expression level of AR mRNA in OSCC cells after EVO treatment was significantly increased (Figure 8A) and (Figure S5). Subsequently, we used SA-β-Gal staining to detect whether EVO induced senescence in CAL27 cells. Compared with the control group, the EVO group exhibited obvious blue staining, increased β-Gal accumulation, and significant cell senescence (Figure 8B). RT-qPCR and Western blotting were used to detect the expression levels of AR, P53, P16, and P21 mRNA and protein in CAL27 cells before and after EVO intervention. After EVO intervention, the mRNA expression levels of P16, P21, and P53 in CAL27 cells were significantly increased (*p* < 0.001, *p* < 0.0001) (Figure 8C(a–c)). The expression levels of P16, P21, and P53 proteins were consistent with their mRNA levels. (Figure 8D). Collectively, the above data indicate that EVO might trigger cellular senescence in OSCC, potentially through the upregulation of AR expression.

## 4. Discussion

As one of the malignant tumors with the highest incidence rate globally, head and neck squamous cell carcinoma (HNSCC) has an annual mortality rate exceeding 500,000 [26]. In recent years, significant progress has been made in the early diagnosis and clinical treatment of HNSCC. Although the introduction of targeted and immunotherapy drugs has improved clinical prognosis to some extent [27], HNSCC patients’ adherence remains poor due to strong toxic side effects and drug resistance, and the 5-year overall survival rate is still unsatisfactory [15]. Therefore, the discovery of new drugs and mechanisms for HNSCC is conducive to the development of potential therapeutic targets, which is of great significance for the treatment of HNSCC.

Isolating effective components from natural medicines and searching for novel anti-tumor drugs among them is a widely accepted anti-tumor strategy. Evodiamine, an alkaloid isolated from the natural medicine *Evodia rutaecarpa*, is one of the main effective components of *Evodia rutaecarpa*. Numerous studies have shown that evodiamine possesses good anti-inflammatory, analgesic, and anti-tumor capabilities. Among them, the excellent anti-tumor ability of evodiamine has attracted the interest of researchers. Studies have shown that EVO can exert its anti-tumor potential by inhibiting cell proliferation, invasion, and migration, inducing cell apoptosis and senescence through pathways such as phosphoinositide 3-kinase/ protein kinase B (PI3K/AKT) and Notch pathways in cancers such as lung cancer, gastric cancer, and oral cancer [4,18]. EVO has also been found to exert its anti-bladder cancer activity by inducing ferroptosis through the inhibition of glutathione peroxidase 4 (GPX4) [19]. Although EVO has shown good anti-tumor effects in oral cancer, its mechanism still needs further investigation.

To further explore the mechanism of EVO’s anti-HNSCC (including OSCC) effect, we regard OSCC as a typical representative of HSNCC and verified the impact of EVO on OSCC cells. Through in vitro experiments, we found that after evodiamine intervention, the proliferation and migration abilities of OSCC cells were significantly inhibited, the cell cycle was significantly arrested, and the ratio of apoptotic cells increased significantly. This result is consistent with previous studies and reports. Additionally, we obtained potential targets of EVO by searching the TCMSP database.

The androgen receptor is a member of the steroid nuclear receptor superfamily, which plays a significant role in normal human physiology and the occurrence and development of various diseases such as tumors [20]. With the continuous updates in AR research, the study of the AR is no longer limited to prostate cancer. Our understanding of its role in cancers such as gastric cancer, lung cancer, liver cancer, and breast cancer is also deepening [21,22,28,29,30]. Studies have shown that the AR plays a dual role in different types of tumors. As a cancer-promoting factor, the abnormal expression of the AR in malignant tumors such as prostate cancer and gastric cancer can promote tumor growth and induce drug resistance in tumor cells [31,32]. Interestingly, however, as a carcinogen in prostate cancer, the continuous activity of the AR can lead to the G1 phase arrest of prostate cancer cells and induce the senescence of cancer cells, thereby exerting an anticancer effect [33]. In estrogen receptor (ER)-positive breast cancer, the overexpression of the AR exerts its tumor-suppressive effect by competitively inhibiting ER signaling through genetic and non-genetic competition [34]. In cervical cancer, the overexpression of the AR significantly inhibits the proliferation of cervical cancer cells [35]. In thyroid cancer, the loss of the AR is closely associated with the progression of thyroid cancer [36]. These studies collectively indicate that the aberrant expression of the AR is not only closely related to the occurrence and development of tumors but also possesses certain tumor-suppressive effects.

While the AR has been extensively studied in prostate cancer, breast cancer, and other malignant tumors, research on its application in head and neck tumors remains scarce. In this study, we conducted a differential gene analysis related to HNSCC based on transcriptome data from 504 HNSCC tissues and 44 normal tissues in the TCGA database, as well as the GSE138206 dataset in the GEO database. Combining the potential targets of EVO in the CellAge database and TCMSP database, we believe that the AR is crucial for our research. We found that the androgen receptor, as a target of EVO, is significantly downregulated in HNSCC tissues. Interestingly, the significant downregulation of AR is significantly correlated with poorer OS outcomes and pathological grading in HNSCC patients. In addition, IHC staining indicated that the expression level of AR in tumor tissues of HNSCC patients is significantly lower than that in paracancer tissues, and the expression of AR in poorly differentiated tumor tissues is significantly lower than that in moderately and highly differentiated tissues, which is consistent with the results of bioinformatics analysis.

Senescence is a ubiquitous phenomenon in multicellular organisms and also serves as one of the fundamental events in tumor suppression. Hayflick [37] first described the senescence phenomenon in cell cultures in vivo, namely replicative senescence, in 1961. Subsequently, stress-induced senescence (SIPS), triggered by the exposure of cells to various stimuli such as reactive oxygen species (ROS), was discovered. In recent years, with the deepening of research on the relationship between cell senescence and tumors, senescent tumor cells have been continuously discovered in tumor cells after chemotherapy. This makes inducing tumor cell senescence through drugs or external physical stimuli to inhibit the proliferation and metastasis of malignant tumors a potential means of treating malignant tumors. Wang [38] found that curcumin can activate the P53/P21 pathway in cervical cancer cells and increase the expression of P53 and P21, leading to irreversible cell cycle arrest and triggering the senescence of cervical cancer cells. Resveratrol can inhibit telomerase activity in ovarian cancer by downregulating the expression level of human telomerase reverse transcriptase (hTERT), inducing tumor cell senescence while also inhibiting its invasive ability [39]. Berberine can induce senescence in glioblastoma cells by inhibiting the epidermal growth factor receptor/mitogen-activated protein kinase kinase/extracellular regulated protein kinases (EGFR-MEK-ERK) pathway, and in colorectal cancer, it can also inhibit telomerase activity by downregulating hTERT, shortening telomere length [40,41]. The above demonstrates the significant advantages of natural drug active ingredients in inducing tumor cell senescence and inhibiting tumor proliferation and metastasis.

Here, through a GO enrichment analysis of differentially expressed genes related to HNSCC, we discovered that cellular senescence plays a significant role in the occurrence and development of HNSCC (including OSCC). By intervening with EVO in OSCC cells, we observed significant morphological changes post-intervention, accompanied by an increase in the activity of β-glucosidase associated with cellular senescence. Additionally, there was a notable elevation in the mRNA and protein levels of P53, P21, and P16, indicating that EVO induces cancer cell senescence and inhibits its activity. As a target of EVO, the AR can also induce tumor cell senescence, and AR-induced tumor cell senescence does not lead to the senescence-associated secretory phenotype (SASP) and its associated pro-cancer effects. In this study, we also found that OSCC cells with sustained AR activity exhibited significant morphological changes, with their proliferation and migration abilities significantly inhibited. The activity of senescence-associated β-glucosidase increased, and the mRNA and protein levels of P53, P21, and P16 all significantly increased, suggesting that sustained AR activity induces senescence in OSCC cells and exerts an anti-tumor effect. Furthermore, we observed a significant increase in the mRNA and protein levels of the AR in OSCC cells post-EVO intervention, indicating a certain interaction between EVO and the AR. EVO may induce senescence in OSCC cells by regulating AR activity, thereby exerting its anticancer effect against OSCC.

Based on the aforementioned research, we have preliminarily demonstrated that evodiamine can inhibit tumor cell activity by inducing cell senescence. Additionally, we have confirmed that the AR, besides its carcinogenic role, can also exert an anticancer effect by inducing cell senescence. Our study may have validated the correlation between low AR expression in HNSCC and poor patient prognosis.

Our studies have highlighted the potential of evodiamine in combating HNSCC (including OSCC) through the induction of cellular senescence and the modulation of AR expression. However, the current research on the anti-HNSCC effects and mechanisms of EVO is not without its limitations. Owing to the challenges associated with procuring tissue samples, our study was limited to examining the anti-OSCC effects of EVO, which is predominant in HNSCC, as well as exploring the mechanistic link between AR expression and cellular senescence. Additionally, the current body of research lacks a detailed exploration of EVO’s in vivo anti-OSCC effects by establishing suitable animal models. Future studies should focus on deciphering the intricate pathways through which EVO modulates AR expression and induces cellular senescence, by employing high-throughput or single-cell sequencing, proteomic analysis, and gene editing techniques to comprehensively profile the molecular changes induced by EVO treatment. In addition, future studies could develop animal models for OSCC to assess the therapeutic potential of EVO and to bridge the gap between laboratory findings and clinical applications.

## 5. Conclusions

In summary, this study has established a preliminary correlation between reduced AR expression in OSCC tissues and poor prognosis, as well as advanced clinical staging in the patients. Additionally, our findings have preliminarily demonstrated that evodiamine may elicit its antitumor effects by promoting cellular senescence in OSCC cells through the regulation of AR expression. These results not only propose AR as a potential therapeutic target for HNSCC (including OSCC) but also reinforce the mechanistic understanding of how evodiamine can initiate tumor cell senescence. Consequently, this research provides fresh insights and novel perspectives on the study of evodiamine in the context of HNSCC (including OSCC).

## Figures and Tables

**Table 1:** Summary of the oligonucleotide primer sequences.

Gene	Forward Primer	Reverse Primer
*GAPDH*	GTGGAGTCCACTGGCGTCT	GTCGAGGAGGCATTGCTGAT
*AR*	GACGACCAGATGGCTGTCATT	GGGCGAAGTAGAGCATCC
*p16*	TGGCTCTGACCATTCTGT	AGCTTTGGAAGCTCTCAG
*p21*	GGGATGAGTTGGGAGGAGG	CGGCGTTTGGAGTGGTAG
*p53*	CGCTTCGAGATGTTCCGAGA	CTGGGACCCAATGAGATGGG

**Table 2:** Potential targets of evodiamine.

ID	Molecule Name	Target Name
1	Evodiamine	Gamma-aminobutyric acid receptor subunit alpha-1
2		Beta-2 adrenergic receptor
3		mRNA of PKA catalytic subunit C-alpha
4		Nuclear receptor coactivator 1
5		Calcium-activated potassium channel subunit alpha 1
6		Muscarinic acetylcholine receptor M3
7		Retinoic acid receptor RXR-alpha
8		Coagulation factor VII
9		Carbonic anhydrase II
10		5-hydroxytryptamine receptor 3A
11		Trypsin-1
12		Nitric-oxide synthase, endothelial
13		Prostaglandin G/H synthase 2
14		Muscarinic acetylcholine receptor M5
15		Phosphatidylinositol-4,5-bisphosphate 3-kinase catalytic subunit, gamma isoform
16		Coagulation factor Xa
17		Sodium channel protein type 5 subunit alpha
18		Prostaglandin G/H synthase 1
19		Heat shock protein HSP 90
20		Androgen receptor
21		Muscarinic acetylcholine receptor M1
22		Potassium voltage-gated channel subfamily H member 2

## Data Availability

These data were derived from the following resources: TCGA (https://www.cancer.gov/ccg/research/genome-sequencing/tcga, accessed on 1 November 2024) and GEO (https://www.ncbi.nlm.nih.gov/geo/, accessed on 6 November 2024), TCMSP (https://www.tcmsp-e.com/#/database, accessed on 10 November 2024), CellAge (https://genomics.senescence.info/cells/, accessed on 12 November 2024), GEPIA (http://gepia.cancer-pku.cn/, accessed on 14 November 2024), HPA (https://www.proteinatlas.org/, accessed on 13 November 2024), UCSC (https://genome-asia.ucsc.edu/index.html, accessed on 13 November 2024), cBioProtal (https://www.cbioportal.org/, accessed on 13 November 2024), TIMER 2.0 (http://timer.cistrome.org/, accessed on 15 November 2024), Kaplan–Meier Plotter (https://kmplot.com/analysis/index.php?p=service&cancer=pancancer_rnaseq, accessed on 15 November 2024), and ShinyThor App (https://alexismurillo.shinyapps.io/ShinyThor, accessed on 28 June 2025).

## Supplementary Materials

The supporting information can be downloaded at https://www.mdpi.com/article/10.3390/cimb47070558/s1.
