# Supplementary material for: Evodiamine Boosts AR Expression to Trigger Senescence and Halt Proliferation in OSCC Cells
Source: Curr Issues Mol Biol. 2025 Jul 17;47(7):558. doi: 10.3390/cimb47070558 (PMC12293151; doi:10.3390/cimb47070558)
Supplement: Supplementary file 1 [file cimb-47-00558-s001.zip › cimb-3701122-supplementary.pdf]

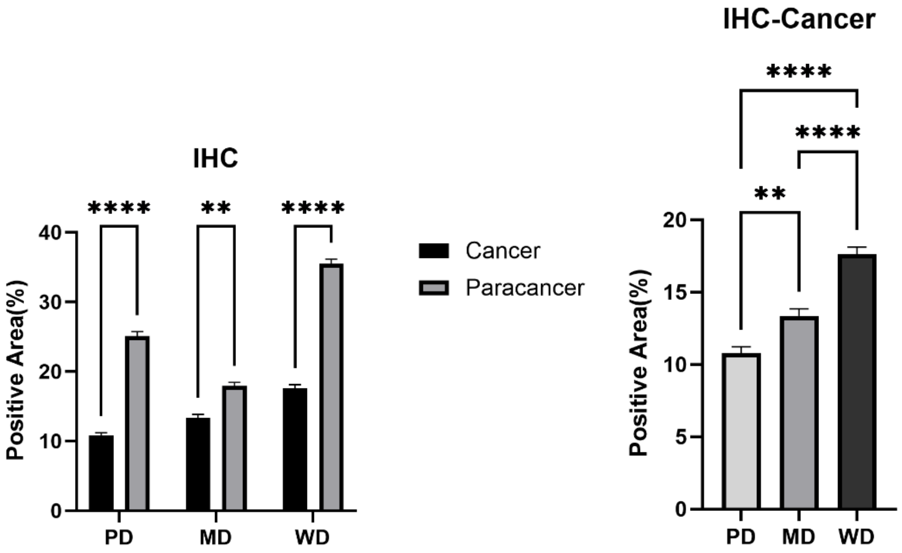

Figure S1. AR expression profiles in tissues of OSCC and adjacent non-cancerous by Immunohistochemical detection. The expression of AR is correlated with the degree of differentiation of cancerous tis-sues, with higher expression levels corresponding to higher levels of differentiation. $*p < 0.05$, $**p < 0.01$, $***p < 0.001$.

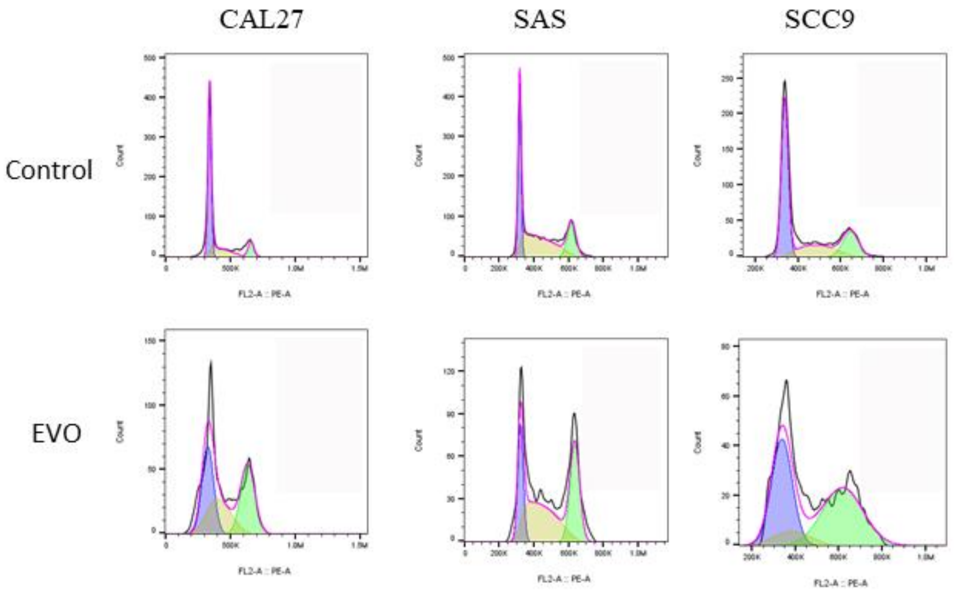

Figure S2. Flow cytometry was used to detect cell apoptosis after 48 hours of EVO treatment, revealing that EVO can induce G2/M phase arrest in OSCC cells.

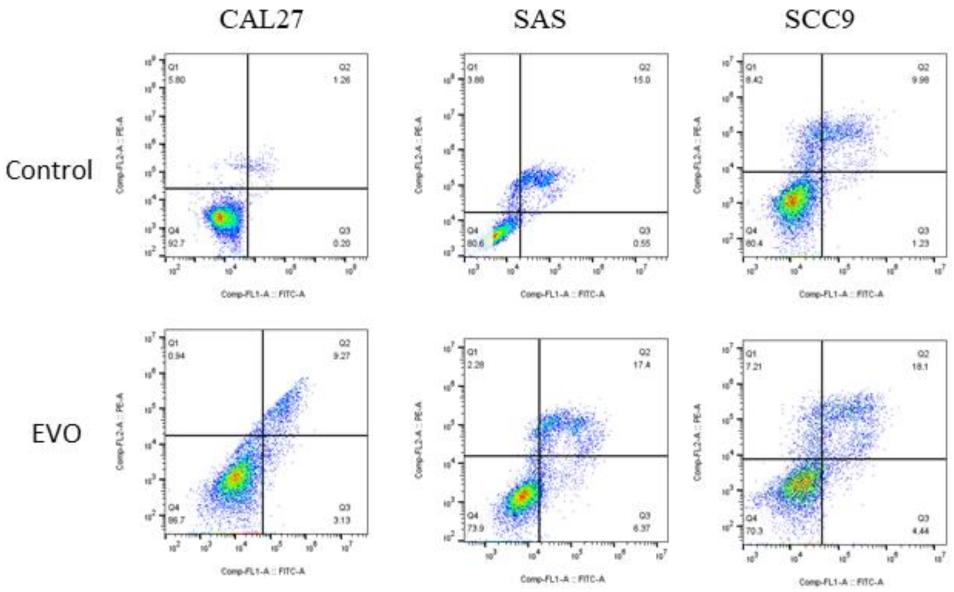

Figure S3. Flow cytometry was used to detect cell apoptosis after 48 hours of EVO treatment, revealing that EVO can induce apoptosis in OSCC cells.

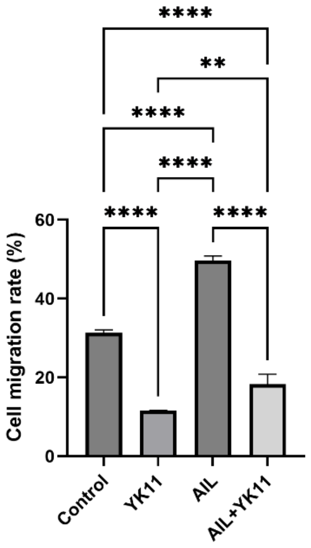

Figure S4. Wound healing assay to evaluate the effect of AR overexpression on the migration ability of CAL27 cells. YK11-induced AR overexpression effectively inhibited the migration ability of OSCC cells. *p < 0.05, **p < 0.01, ***p < 0.001.

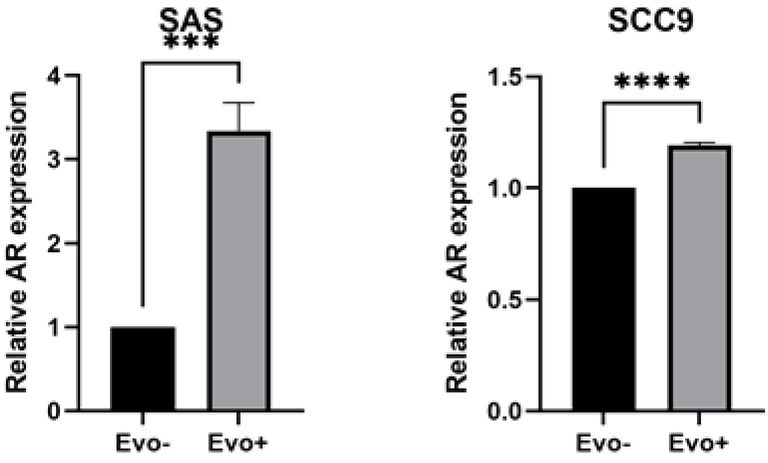

Figure S5. EVO treatment of OSCC cells significantly increases the expression level of AR mRNA. *p < 0.05, **p < 0.01, ***p < 0.001.

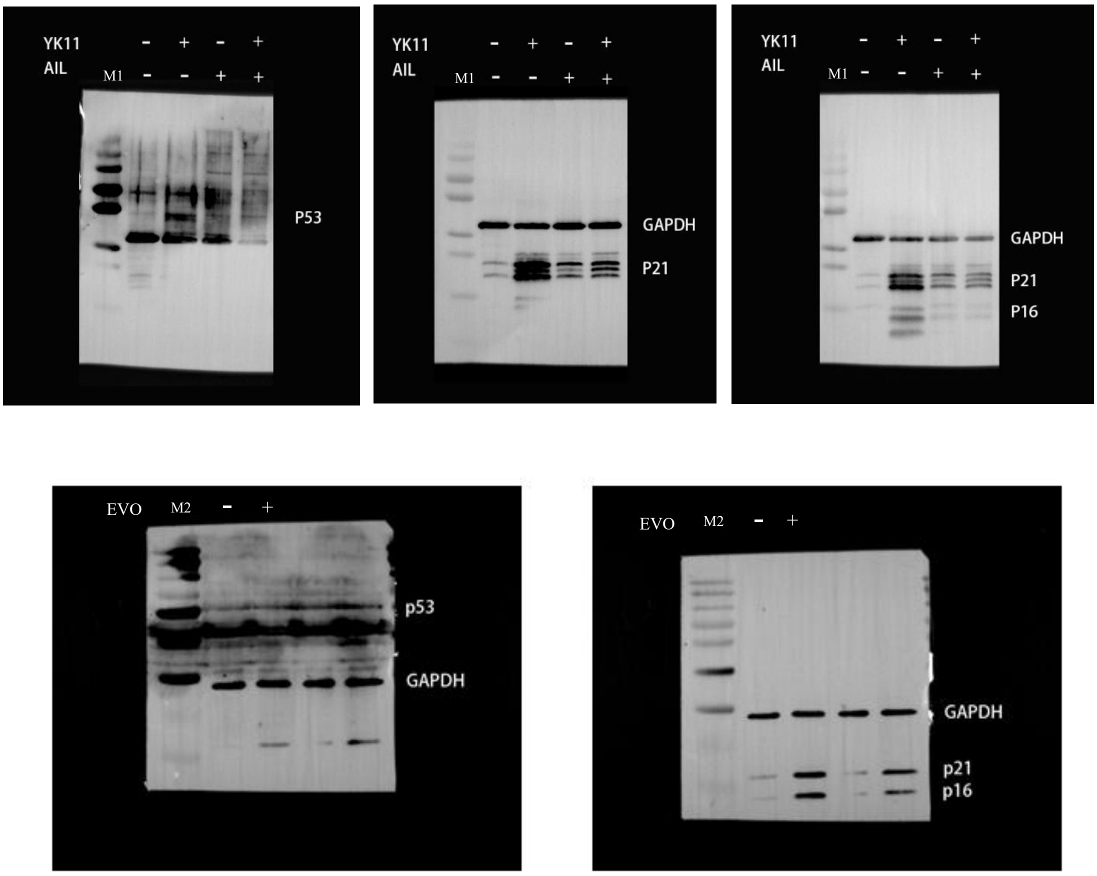

Figure S6. Full-length blots/gels of Figure 7J and Figure 8D. Protein Ladder M1(Cat. SB-26619, 8-250kDa) were provided by Sharebio (Shanghai, China), Protein Ladder M2(Cat. G2083-250UL, 8-200kDa) were provided by Servicebio (Wuhan, China).

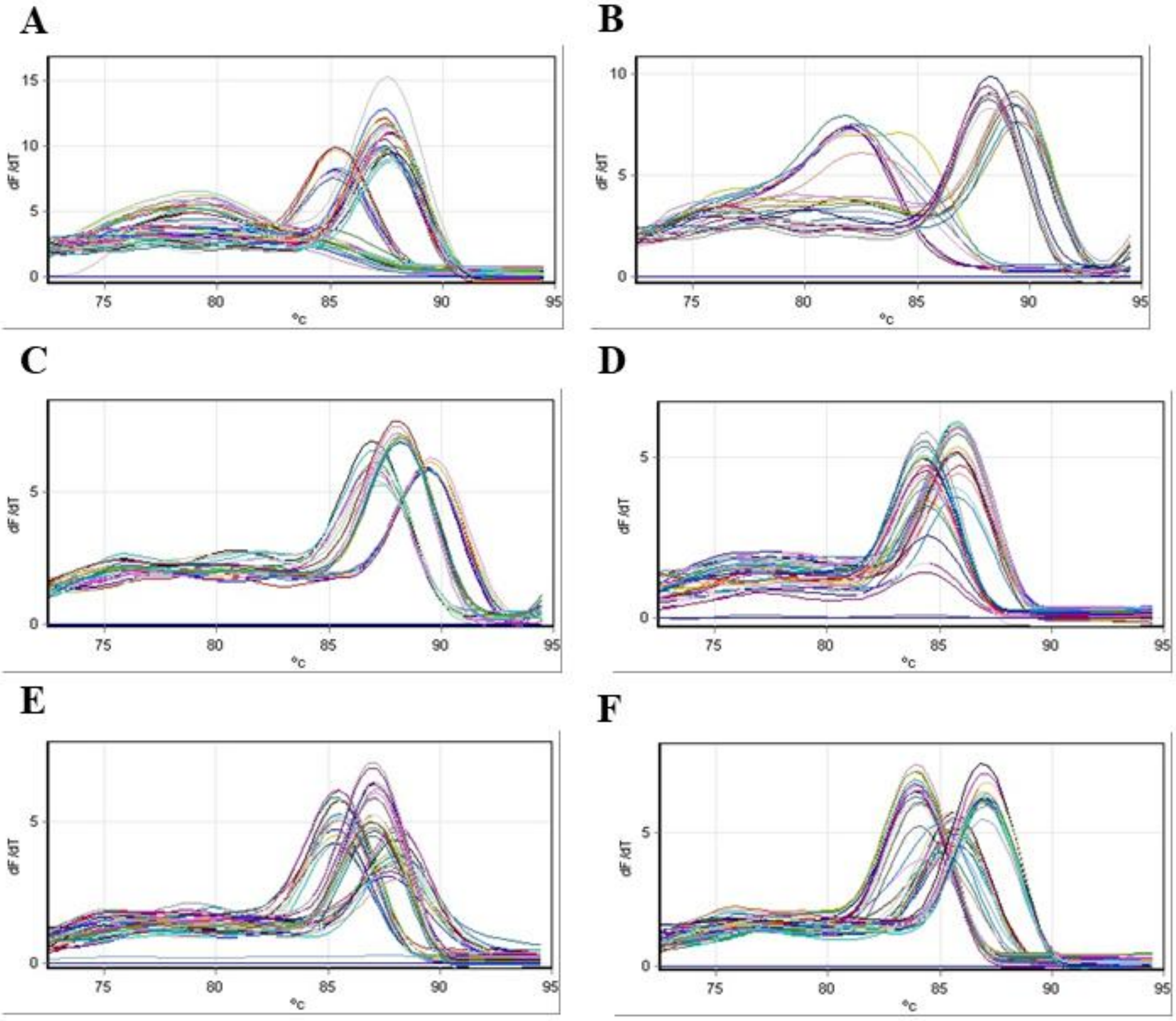

Figure S7. Melting curves of RT-qPCR. (A) GAPDH and AR (Control vs EVO). (B) GAPDH, p16 and p53 (Control vs EVO). (C) GAPDH, p21 and p53 (Control vs EVO). (D) GAPDH and AR (Control vs YK11 vs AIL vs AIL+YK11). (E) GAPDH, p16 and p53 (Control vs YK11 vs AIL vs AIL+YK11). (F) GAPDH, p21 and p53 (Control vs YK11 vs AIL vs AIL+YK11).

Table S1. GO and KEGG enrichment analysis results (TOP5)

ONTOLOGY	ID	Description	GeneRatio	BgRatio	pvalue	p.adjust	qvalue	Count	zscore
BP	GO:0007346	regulation of mitotic cell ...	70/431	478/18800	2.76E-36	1.38E-32	6.90E-33	70	2.1514
BP	GO:0044772	mitotic cell cycle phase tr...	65/431	440/18800	6.51E-34	1.62E-30	8.14E-31	65	3.1009
BP	GO:0050673	epithelial cell proliferation	61/431	443/18800	4.41E-30	7.32E-27	3.67E-27	61	-0.12804
BP	GO:0045786	negative regulation of cell...	55/431	387/18800	7.36E-28	9.18E-25	4.60E-25	55	2.2923
BP	GO:1901990	regulation of mitotic cell ...	50/431	321/18800	2.95E-27	2.94E-24	1.47E-24	50	3.1113
CC	GO:0005667	transcription regulator com...	52/433	483/19594	2.10E-21	9.30E-19	6.47E-19	52	1.3868
CC	GO:0005819	spindle	34/433	402/19594	2.38E-11	5.25E-09	3.65E-09	34	3.43
CC	GO:0090575	RNA polymerase II transcrip...	25/433	230/19594	5.86E-11	8.08E-09	5.62E-09	25	-0.2
CC	GO:0005925	focal adhesion	34/433	419/19594	7.32E-11	8.08E-09	5.62E-09	34	2.401
CC	GO:0017053	transcription repressor com...	15/433	76/19594	9.54E-11	8.44E-09	5.87E-09	15	0.2582
MF	GO:0004712	protein serine/threonine/ty...	57/432	446/18410	6.23E-26	4.41E-23	3.36E-23	57	1.7219
MF	GO:0140297	DNA-binding transcription f...	58/432	470/18410	1.39E-25	4.93E-23	3.75E-23	58	0.26261
MF	GO:0004674	protein serine/threonine ki...	49/432	430/18410	3.34E-20	7.89E-18	6.00E-18	49	2.1429
MF	GO:0106310	protein serine kinase activity	44/432	360/18410	2.07E-19	3.66E-17	2.79E-17	44	1.8091
MF	GO:0061629	RNA polymerase II-specific ...	43/432	348/18410	3.58E-19	5.07E-17	3.86E-17	43	0.76249
KEGG	hsa04218	Cellular senescence	42/319	156/8164	2.05E-24	5.64E-22	3.35E-22	42	2.1602
KEGG	hsa04110	Cell cycle	38/319	126/8164	4.03E-24	5.64E-22	3.35E-22	38	2.5955
KEGG	hsa05161	Hepatitis B	35/319	162/8164	3.86E-17	3.60E-15	2.14E-15	35	1.5213
KEGG	hsa05215	Prostate cancer	27/319	97/8164	2.01E-16	1.40E-14	8.34E-15	27	-0.57735
KEGG	hsa05167	Kaposi sarcoma-associated h...	36/319	194/8164	2.25E-15	1.00E-13	5.96E-14	36	1

Table S2. AR expression in upper_aerodigestive_tract cell lines

ID	Pathology ID	Tissue differentiation grade	TNM stage
1	66259582	Poorly differentiated	T1N2bM0
2	66263633	Poorly differentiated	T3N2bM0
3	66264105	Well differentiated	T2N2bM0
4	66265712	Moderately differentiated	T2N2bM0
5	66266078	Well differentiated	T2N0M0
6	66266134	Moderately differentiated	T2N1M0
7	66266342	Poorly differentiated	T2N1M0
8	66272818	Well differentiated	T1N0M0
9	66500405	Moderately differentiated	T2N0M0
10	66504510	Poorly differentiated	T2N0MX

Table S3. AR expression in upper_aerodigestive_tract cell lines

ID	DepMap ID	Cell line	ARlog2(TPM+1)
1	ACH-000715	SNU-1214	0.73
2	ACH-000832	CAL 27	0.41
3	ACH-000682	SNU-1066	0.28
4	ACH-000846	FaDu	0.25
5	ACH-000503	BICR 16	0.18
6	ACH-000500	SNU-46	0.11
7	ACH-000735	PE/CA-PJ49	0.07
8	ACH-000546	HSC-4	0.06
9	ACH-000630	YD-8	0.04
10	ACH-000692	SNU-899	0.04
11	ACH-000762	YD-38	0.04
12	ACH-000238	SCC-4	0.03
13	ACH-000518	CAL-33	0.03
14	ACH-000548	BHY	0.03
15	ACH-000723	YD-10B	0.03
16	ACH-000181	SCC-9	0.01
17	ACH-000228	BICR 31	0.01
18	ACH-000549	SNU-1076	0.01
19	ACH-000618	SNU-1041	0.01
20	ACH-000771	BICR 56	0.01
21	ACH-000188	SCC-25	0
22	ACH-000254	SCC-15	0
23	ACH-000415	BICR 6	0
24	ACH-000472	HSC-2	0
25	ACH-000606	PE/CA-PJ34 (clone C12)	0
26	ACH-000619	PE/CA-PJ15	0
27	ACH-000732	PE/CA-PJ41 (clone D2)	0
